# Stray Thoughts about Regulating

**Published:** 1902-10

**Authors:** S. E. Davenport

**Affiliations:** New York, N. Y.


### STRAY THOUGHTS ABOUT REGULATING.[1]

BY S. E. DAVENPORT, M.D.S., D.D.S., NEW YORK, N. Y.

Not because I have anything new to divulge about the regulation of teeth do I appear before you to-night, but because of pressure brought to bear upon me by our board of directors, who thought it time for me to do something.

When I first began giving attention to the regulation of teeth, so hopeful was my disposition that I fully expected some day either to be told by some experienced *confrère,* or to discover for myself, a series of "underlying principles" so governing the movement of teeth that an understanding of them would enable me to apply the proper rule and consequent appliance to every case, thereby securing an infallibly successful result with but little trouble to the patient or myself.

In the mean time, while waiting for the above described revelation to arrive, I blundered along as well as I could, taking from all

---

[1] Read before The New York Institute of Stomatology, April 1, 1902.

men who wrote about regulating, and from all whom I could hear talk about it, such ideas and points as seemed to me to be practical, changing some for the exigencies of individual cases and deriving more, I am sure, from these writers and speakers than from any originality which I was able to bring to bear.

I regret that I am unable now, after twenty-five years of some experience with this kind of work, to give a list of those principles which in my youthful days I expected some time to acquire; but while I am a little more versed in the possibilities of tooth movement than I was, and even in its probabilities, I am still looking and longing for fixed principles, a knowledge of which would enable us to eliminate even partial failure from our results and to undertake the most difficult cases without much preliminary study.

While there are numerous well-fixed laws governing the movement of teeth and the changes in their environment most helpful to the student of orthodontia, I have been unable to learn of many rules applicable to all classes of cases which would enable one to describe methods based upon such rules with the words "regulation made easy."

In spite of what has just been said, my opinion is that, allowing for differences in the density of the bones and other like variable factors, probably no part of our work is more satisfactory or more stable of logical results if it be approached from the proper direction, one step after another of a carefully devised plan being taken.

I remember well a pet remark of Dr. Bogue's so applicable to this thought as to bear repetition,—"Do it, don't do *at* it!"

The regulation of teeth is one of the most important branches of dental practice, but it seldom receives the attention it deserves even by those who attempt to pursue it.

Hastily formed opinions are often as hastily expressed; spasmodic effort is made to move certain teeth often before the dentist has decided upon his general plan, the result being discouragement of all concerned and a resolution made more firm in the mind of the operator by each failure never to attempt regulating unless forced to.

A proper diagnosis of the needs of each case requires not only a definite appointment for examination of the child's mouth, but the taking of impressions at that sitting and a postponement of the decision as to the plan to be followed until the casts are completed and studied.

The patient should then be seen again and the decision arrived at from the study of the cast tested by a further study of the patient's face.

I wish to emphasize the necessity of studying the face and the plaster cast together. Neither is sufficient, in my opinion, without the other as a basis for a plan, and if a cast were to be shown to me to-day in consultation, while I might give an idea as to the mechanical possibilities, it would not be safe for any one to follow the suggestion unless the probable result would coincide with the needed change in expression of the child's face.

No final advice should be given until the procedure as described above has been gone through with, errors of judgment being much less frequent when a study is made of the teeth, the face, and the plaster cast all at one time.

When the cast is made, the view of the lingual surfaces of the teeth and their occlusion gives us a great advantage over the study of the mouth alone, unable as we are to see the inner surfaces of the teeth when closed.

One of the principal reasons for advising thorough study of a properly articulated plaster cast of every case before the formation of a plan is that extraction would thereby be dispensed with. The avoidance of extraction is desirable in nearly every case, therefore never advise extraction in response to impulse.

The many objections to extraction have been ably brought out in the writings of Dr. Isaac B. Davenport, of Paris, and Dr. E. A. Bogue, of New York, and it is not the province of this paper to mention those objections in detail, but one point that I wish to emphasize is that extraction not only produces the lamentable conditions described by the authors referred to, but actually increases the difficulties the dentist has to contend with when striving to regulate teeth.

I believe it safe to say that wherever nature's laws are broken our task is made greater. Without extraction we have in the crowded arch only to expand the arch and rearrange the teeth. Extraction makes the condition at once abnormal. Many forces of retrogression are started, and in place of simply assisting nature we are obliged to combat her. Frequently cases which appear on first examination to indicate the need for extraction of one or more teeth prove later the error of such judgment, for when the arch is sufficiently expanded for the needs of the other arch and for facial expression the full number of teeth often proves insufficient to fill the space secured.

The casts exhibited illustrate this point, number one showing the condition as the teeth were when a gentleman who practices in New York recommended the extraction of a superior bicuspid for the purpose of, in his opinion, simplifying the process of regulation. Cast number two shows the case four months later, unfinished, but with the upper arch expanded and, as will be seen, too much room existing even without extraction.

The time being insufficient for considering further the many objections to extraction, I will refer to some methods for making the patient's suffering as light as possible.

My experience has taught me that in both regulating and wedging the suffering is caused almost entirely by interference with the gum, for teeth can be moved a considerable distance with but little pain or soreness if the appliance or material used for the purpose can be kept away from the gum.

This fact accounts largely not only for the great suffering consequent upon the old methods of wedging when rubber and wood were generally used, but also to a degree for the lessened pain from wedging with tape and for the almost entire absence of discomfort even from wedging with fish-line or other suitable cord tied between the teeth.

The great advantage of the last-mentioned method of wedging is that the more the material swells—and some of the most tightly twisted continue to swell for more than forty-eight hours—the further away from the gum it is.

So great a change has this method of wedging made in my practice that many patients who previously dreaded and suffered from the wedging more than the subsequent operations now assure me that they would hardly know the teeth were being wedged were it not for the detection by the tongue of the little knot or ends of the line.

This comment upon wedging materials is not so much of a digression from the subject of this little paper as it at first seems, inasmuch as the use of fish-line wedges constitutes a considerable part of many minor cases of regulating.

Dr. Isaac B. Davenport, of Paris, was, so far as I know, the first to make use of fish-line for wedging.

At the May, 1899, meeting of the Institute it was my privilege to describe, with some attention to detail, Dr. Davenport's methods of adjusting the fish-line and the result of my own experience with it for quite a number of years.

The record of this can be found in the *International Dental Journal,* September, 1899, pages 586, 587.

Because of what I have learned, considering the advisability of keeping away from the gum as much as possible, I have for a long time depended almost entirely upon fixtures easily removable, relying principally upon springs and screws for the power of action.

At first glance this would seem to many bad generalship, enabling the patient, as it does, at any time to remove the fixture, but this apparent disadvantage amounts to nothing in comparison with its many advantages when one refuses, as I think all practitioners should, to undertake a case of regulating unless sure of the earnest co-operation of the patient, or at least of the one having most influence with the patient.

Among the advantages possessed by removable fixtures are the possibility of keeping both the teeth and fixture clean, the lack, as above stated, of uncomfortable impingement upon the gum, the freedom of the patient's mouth at meals from the appliance and the consequent enjoyment of food with lessened interference with nutrition, and last but not least, the absence of that feeling of utter hopelessness which a secured fixture usually gives a child, resulting often in not only a lessened interest in the result, but sometimes in a desperate disregard of all appeals for co-operation.

Acknowledging the rule of exceptions, there are, of course, occasional cases necessitating the opening of the bite, in which the patient must wear an appliance during meals, removing it only for the purpose of cleansing, but wherever possible, and in spite of a knowledge of strong advantages possessed by certain fixed systems of regulating appliances, such as the Angle, Knapp, etc., I should advise the use of removable appliances if there were no other reason than the greater health of the mucous membrane which almost always accompanies their use.

It is not necessary for me to even remind my hearers that regulating plates must not extend over the masticating surfaces of the back teeth unless a shortening of the bite is desired, and fortunately, since Dr. V. H. Jackson, of New York City, so completely systematized the use of wire cribs and half-cribs, we are able to adjust to the teeth fixtures accurately and securely without shortening the bite to any great extent.

Too much credit cannot be given to Dr. Jackson for all he has done for the great department of orthodontia, and while, of course, we may at times prefer some modifications of the fixtures which he so strongly advises the use of, his inventive genius and free contributions to our knowledge have placed the whole profession under great obligation to him.

### EXPANSION OF THE UPPER ARCH.

While I have successfully used the Coffin plate and modifications of it for expanding the upper arch, which, by the way, is one of the most frequent phases of regulating we are called upon to perform, for a number of years I have in almost every case used a rubber plate, split part way through, with a gold and platinum or platinum and iridium screw, made in my own laboratory, for the motive power, and so arranged that if advisable the patient may take charge of the expansion.

As this is not a double-ended screw, the pressure and consequent movement of teeth is slightly unequal, and, as every arch needs greater expansion on one side than on the other, this unevenness of action is an advantage, for one can always so place the screw when making the plate as to give the greatest movement to the side requiring it.

The position of the screw in these split plates and the length of the split govern the teeth moved and the amount of movement effected.

These split expansion plates are often valuable for use after sufficient expansion has been secured, not only as temporary retainers, but to hold little screws which are placed in the rubber behind individual teeth needing more movement in some direction.

For this purpose German silver wire of various sizes cut in a coarse thread screw-plate answers as well as anything, with the advantage of being inexpensive.

German silver is a very valuable aid in regulating, it being suitable for the Jackson cribs and half-cribs, for spring wires when the greatest force is not needed, for use in rubber fixtures where strength without bulk is desirable, and, as has already been said, for the screws so often needed in the edge of rubber fixtures.

German silver does not answer for the expansion screws or any designed to run in metal nuts, for the material does not possess sufficient strength to make its thread durable when in contact with metallic surfaces.

While referring to German silver and its uses in regulating, it may not be amiss to remind my hearers that it is very useful in other ways to the dentist, inasmuch as it can be soldered with eighteen-carat solder without injury, is not affected by contact with fresh amalgam or even pure mercury, and rubber vulcanizes about it almost as well as around gold or platinum, provided the German silver has been kept from the tarnishing influence of the atmosphere, and even then if the tarnished surface be removed with sand-paper or a file.

There are two kinds of German silver, and for dental purposes the hard and springy variety is desirable. It is procurable of Patterson Bros., 27 Park Row.

### OPENING THE BITE.

The expansion of the back part of a lower arch is usually most readily effected by a plate made after the Coffin principle, using either piano wire, German silver, or platinoid for the spring connecting the two parts of the plate.

If a general expansion of the front part of the arch is desirable, it may be effected either by the use of Dr. H. A. Baker's plan, which is a rubber plate the upper portion of which is split as far back on each side as the teeth need to be moved, an ingeniously bent spring of piano wire or platinoid furnishing the power; by a rubber plate split somewhat as in the Baker plate, but with a gold and platinum jack-screw instead of a spring; or by a Jackson plate or rubber modification with German silver or platinoid springs.

When certain lower teeth only need to be moved out, an ordinary rubber plate so constructed that the closing of the jaws will cause pressure may be used, the little German silver screws being placed in the rubber behind the offending teeth and turned out a little daily with pliers or a watch-key.

I have said so much about the use of these little screws in rubber plates, and I find them so valuable, that it may be best to say a few words regarding their application.

The size of screw desired having been chosen, it is measured in the bur gauge and a bur one size smaller than the screw is placed in the engine ready for use.

The rubber plate then being in position in the mouth, the angle desired is marked with a lead-pencil upon the rubber, the plate then being removed and drilled for the screw, which is turned into its rubber socket with a small hand-vice.

Often the screw will need to entirely penetrate the plate in order that some material may be ready to be turned out against the tooth, and until it is turned out the exposed end should be covered with soft gutta-percha or composition to protect the tongue.

Usually in regulating the dentist will think of more than one way of doing the same thing, and if so, it is not only his duty but for his interest, and will make for the success in the case, to choose the method least uncomfortable for the patient and the least objectionable in appearance.

OPENING THE BITE.

One of the most frequent defects which we are called upon to treat is that class of so-called short bite cases in which the lower incisors bite almost into the gum behind the upper incisors. This condition is caused by a variety of influences, among which I will mention extraction, tardy development of some of the back teeth, and previous attempts at regulation by the use of plates capping the teeth.

Oftentimes the correction of this condition is the key to the solution of all the problems presented by that mouth, and is therefore the first trouble to be overcome.

It is generally corrected by a thin rubber plate in the roof of the mouth held up by atmospheric pressure or by light half-cribs, the lower incisors and cuspids biting against the rubber behind the upper teeth in such manner as to open the bite and leave the molars and bicuspids without occlusion.

Not only does elongation of these back teeth naturally follow, but the lower six front teeth, receiving as they do the entire force of the bite, are slowly but perceptibly shortened, and often in a few weeks the back teeth with the plate in position and the mouth closed will be in firm contact.

Should the amount of change effected be insufficient, an addition of rubber to the part of the upper plate against which the lower front teeth strike will result in an added elongation of the back teeth. Dr. C. A. Woodward, of New York City, an expert in orthodontia, has carried to successful conclusion many important cases of this character, and treats them by placing temporary gold crowns upon the lower bicuspids.

This method has the advantage of relieving the patient of a plate, and never causes any forward movement of the upper front teeth, which does occasionally follow the use of a plate in the roof of the mouth, but it has the disadvantage, it seems to me, not only of failing to shorten the lower incisors and cuspids, sometimes so necessary, but, the great pressure of mastication being borne entirely by the bicuspids, usually shortens them materially.

While nature usually allows these bicuspids to elongate quickly when the gold crowns, having effected their object, are removed. I confess a preference for either the plate method or a combination of the two, in spite of the success attending my use of Dr. Woodward's method in two or three cases.

Some one has said, "Fortunate is the man who lacks inventive genius, for he is able without prejudice to choose the best ideas of all men."

This seems to me to be in a measure true in influencing success in regulating, the widely varying demands of the different cases presenting requiring the use of many principles and forces.

One possessing great powers of invention is not only apt to have his ideas running in a groove, but is likely to depend too much upon himself for plans and methods.

Still, there is no department of our specialty, probably, which so successfully trains one of even ordinary ability to think and act quickly and logically as does orthodontia.

It has always seemed to me, therefore, that those dentists who decide not to accept or "bother with," as the phrase goes, regulating cases deny themselves the advantages of one of the best possible training schools in which patience, quick thought, and readiness for emergencies are taught more effectually than by any other department of our work.

Regulation of the teeth also fills the longing felt by many of us for action outside of ordinary dental practice, giving sufficient diversity to our work to prevent the mental inertia which is so apt to come to the professional man who is called upon to do practically the same things each day.

